# A Mixed-Micelle Capillary Electrophoresis Method for Fast Quality Screening of Antimalarial Drugs

**DOI:** 10.3390/molecules31213884

**Published:** 2026-10-28

**Authors:** Hailekiros Gebretsadik Kidanemariam, Erwin Adams, Ann Van Schepdael

**Affiliations:** 1 Pharmaceutical Analysis, Department of Pharmaceutical and Pharmacological Sciences, KU Leuven—University of Leuven, Herestraat 49, O&N2, PB 923, 3000 Leuven, Belgium; hailekiros.kidanemariam@kuleuven.be (H.G.K.); erwin.adams@kuleuven.be (E.A.); 2 Faculty of Pharmacy, Pharmaceutical Analysis and Quality Assurance, College of Health Sciences, Mekelle University, Mekelle P.O. Box 231, Ethiopia

**Keywords:** mixed-micelle MEKC, antimalarial drugs, quality screening, falsified medicines, substandard medicines, resource-limited settings

## Abstract

Substandard and falsified antimalarial medicines remain a major barrier to effective malaria treatment, particularly in resource-limited settings where accessible and reliable analytical tools are essential for medicine-quality screening. To support this need, we developed a mixed-micelle micellar electrokinetic chromatography (MEKC) method for the simultaneous analysis of five antimalarial drugs. The optimized background electrolyte consisted of 10 mM borate buffer, 55 mM lithium dodecyl sulfate (LiDS), 10 mM polyoxyethylene (23) lauryl ether (Brij-35), and 30% acetonitrile at pH 8. Under these conditions, all analytes were baseline-resolved within a total analysis time of less than 7 min, showing good peak symmetry and repeatable separation. The method also consistently resolved minor impurity peaks present in quinine and primaquine standards, demonstrating its capability to detect additional components relevant to quality surveillance. Method validation demonstrated adequate selectivity, repeatability, robustness, linearity, and sensitivity. The method was successfully applied to commercial antimalarial medicine samples, demonstrating its potentiality for rapid quality screening of pharmaceutical formulations. Overall, the mixed-micelle MEKC approach offers a rapid, reliable, and affordable tool for the simultaneous screening of antimalarial medicines, with strong potential for use in resource-limited settings.

## 1. Introduction

### 1.1. The Burden of Substandard and Falsified Antimalarial Medicines

Malaria remains one of the world’s most devastating infectious diseases, killing hundreds of thousands of people each year and placing a disproportionate burden on communities across Sub-Saharan Africa [1,2]. Over 90% of global malaria cases and deaths occur in low- and middle-income countries (LMICs), where young children and pregnant women represent the most vulnerable groups [1,3]. Although the introduction of artemisinin-based combination therapies and long-standing antimalarial agents has substantially reduced malaria mortality, these gains are increasingly threatened by the widespread circulation of substandard and falsified antimalarial medicines [4,5,6].

Studies indicate that approximately 19% of antimalarials in LMICs fail quality standards or contain insufficient or no active pharmaceutical ingredients [4]. Substandard antimalarial formulations can lead to therapeutic failure, allowing otherwise uncomplicated infections to progress into severe, life-threatening disease, including cerebral malaria [6,7,8]. Inadequate drug exposure also accelerates the emergence and dissemination of drug-resistant *Plasmodium* strains, notably variants associated with artemisinin resistance [9,10]. Moreover, poor-quality antimalarials impose significant economic burdens on households, who often incur repeated costs for ineffective treatments, face preventable medical complications, and lose income due to prolonged illness [7,11,12]. These realities highlight the urgent need for rapid, robust, and affordable quality-control approaches that can detect compromised formulations before they reach patients.

### 1.2. Analytical Challenges in Low-Resource Settings

Robust post-marketing surveillance systems are essential for ensuring the quality of pharmaceuticals. However, in many resource-limited countries, financial constraints, inadequate infrastructure, and shortages of trained personnel continue to hinder their effective implementation and operation [13]. Although analytical techniques such as LC–UV and LC–MS provide robust and reliable quantitative analysis, their long-term implementation can be difficult to sustain in low-resource settings. The high costs of purchasing and maintaining these instruments, the need for stable electricity, dependence on continuous solvent supplies, and delays in obtaining technical assistance make routine use challenging and lead to major gaps in medicine-quality surveillance.

To address these constraints, simplified field screening tools such as the Global Pharma Health Fund Minilab have been widely adopted. Combining visual inspection, colorimetric assays, and thin-layer chromatography (TLC), these kits offer portability and low operating cost [14,15]. However, TLC provides primarily qualitative or semi-quantitative information and offers limited chromatographic resolution. Its capacity to resolve structurally similar components, quantify low-level degradation products, or detect trace impurities is limited. These limitations highlight the need for an alternative analytical approach that maintains the affordability and low solvent consumption of field kits while delivering the quantitative reliability and selectivity of advanced techniques. In this study, we employ micellar electrokinetic chromatography (MEKC) as a practical solution for the quality screening of five widely used antimalarial agents. Because capillary electrophoresis (CE) techniques can operate in multiple modes, they are suitable for analysing a broad range of molecules. Notably, MEKC extends this versatility by enabling the separation of neutral analytes through micelle-mediated partitioning.

### 1.3. Review of Electrophoretic Approaches for Antimalarial Drugs

Various CE techniques including CZE and MEKC have been employed for the analysis of antimalarial drugs in pharmaceutical formulations and biological matrices. Studies have described the application of CZE and other CE modes to the analysis of antimalarial drugs such as chloroquine, quinine, piperaquine, amodiaquine, amodiaquine/artesunate, pyrimethamine, and pyrimethamine/sulfadoxine [16,17].

MEKC provides an alternative approach when differences in hydrophobicity and interactions with the pseudostationary phase can be exploited to improve separation selectivity. An MEKC method was developed for the simultaneous determination of artesunate and amodiaquine in fixed-dose combination tablets [18]. In another study, an MEKC method was proposed for the separation of mixtures of antimalarial drugs. The method was applied for the analysis of antimalarial medicines obtained from the African market [19].

Other electromigration approaches have also been reported. Microemulsion electrokinetic chromatography was used for the simultaneous determination of artemether and lumefantrine in fixed-dose combination tablets, demonstrating the usefulness of alternative electrokinetic separation systems for poorly water-soluble antimalarials [20]. CZE has been applied to amodiaquine and its related impurities [21] and to primaquine in pharmaceutical formulations using multiple- and short-end-injection strategies [22]. MEKC coupled with MS has also been employed for the determination of artemisinin and related compounds in *Artemisia annua* extracts [23].

**Figure 1:**
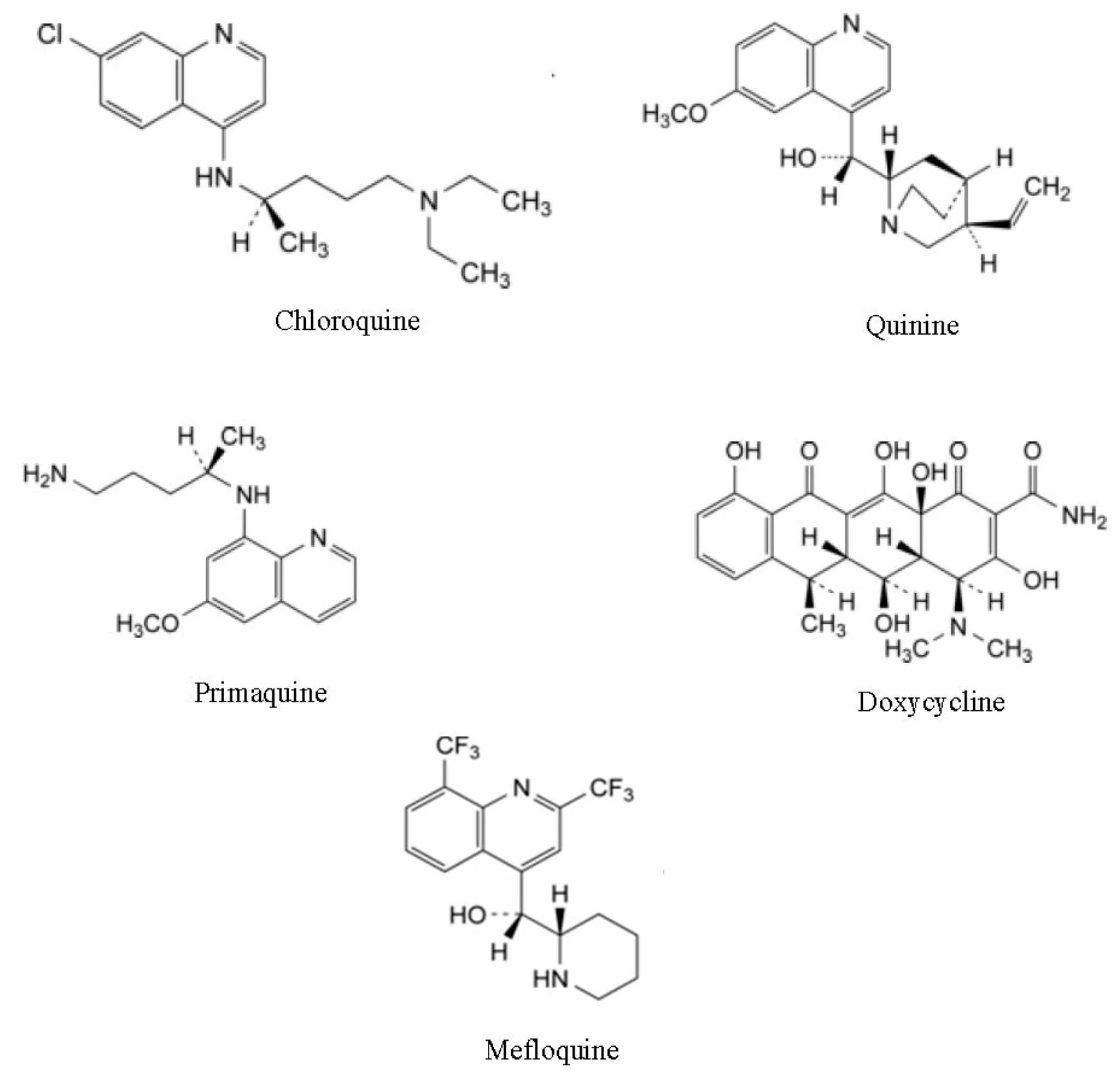
Chemical structure of studied antimalarial drugs (Ph. Eur.).

In the present study, a mixed micellar system composed of lithium dodecyl sulfate (LiDS) and polyoxyethylene (23) lauryl ether (Brij-35) was employed for the simultaneous analysis of chloroquine, doxycycline, primaquine, quinine, and mefloquine (Figure 1). The inclusion of doxycycline, which is used for malaria chemoprophylaxis, extends the analytical scope beyond conventional antimalarial drug combinations and encompasses compounds relevant to both malaria prevention and treatment. The use of sodium dodecyl sulfate (SDS) or LiDS alone resulted in prolonged retention of the analytes, prompting the incorporation of the non-ionic surfactant Brij-35 to modify the separation environment and facilitate the simultaneous analysis of these structurally diverse compounds with different physicochemical and ionization properties. The use of mixed LiDS/Brij-35 micelles provides an additional means of controlling separation selectivity compared with LiDS or SDS alone. While SDS or LiDS forms the charged micellar phase required for conventional MEKC, incorporation of the non-ionic surfactant Brij-35 can modify analyte–micelle interactions and, consequently, migration behaviour and selectivity [24]. These effects have also been demonstrated in pharmaceutical analysis, where the addition of Brij-35 to SDS-based micelles produced changes in migration behaviour and separation selectivity [25]. Mixed-micellar systems have continued to be recognized as a useful strategy in CE for modifying separation selectivity and electroosmotic flow (EOF) [26].

## 2. Results and Discussion

### 2.1. Method Development and Systematic Optimization

The objective of this study was to develop a rapid, cost-effective, and robust MEKC method suitable for screening antimalarial formulations in resource-limited settings. Because the target antimalarial drugs have a wide range of physicochemical properties, MEKC was selected as the most appropriate CE mode for simultaneously resolving the five selected antimalarial drugs and the internal standard. The use of a surfactant-based micellar phase enabled efficient separation of neutral, acidic, and basic analytes within a single run. To achieve optimal resolution, key electrophoretic parameters, including buffer composition, surfactant type and concentration, organic modifier content, and background electrolyte (BGE) pH, were systematically evaluated and optimized. This comprehensive optimization allowed reliable separation of all five antimalarial drugs simultaneously.

Wavelength optimization was performed during method development using the multi-wavelength viewing function in the software of the CE equipment. A wavelength of 220 nm was selected for the study because it provided the best balance between sensitivity, signal quality, and baseline stability across all analytes.

#### 2.1.1. Background Electrolyte Screening

The initial phase of method development focused on establishing a robust MEKC system capable of separating three antimalarial agents, doxycycline, chloroquine, and quinine, with subsequent expansion to include primaquine and mefloquine. SDS was evaluated as a surfactant in combination with phosphate and borate buffer systems. These buffers were selected for their effective buffering capacity in the alkaline pH range, where the analytes exhibit a distinct ionisation state which is expected to enhance electrophoretic selectivity and facilitate their separation.

**Figure 2:**
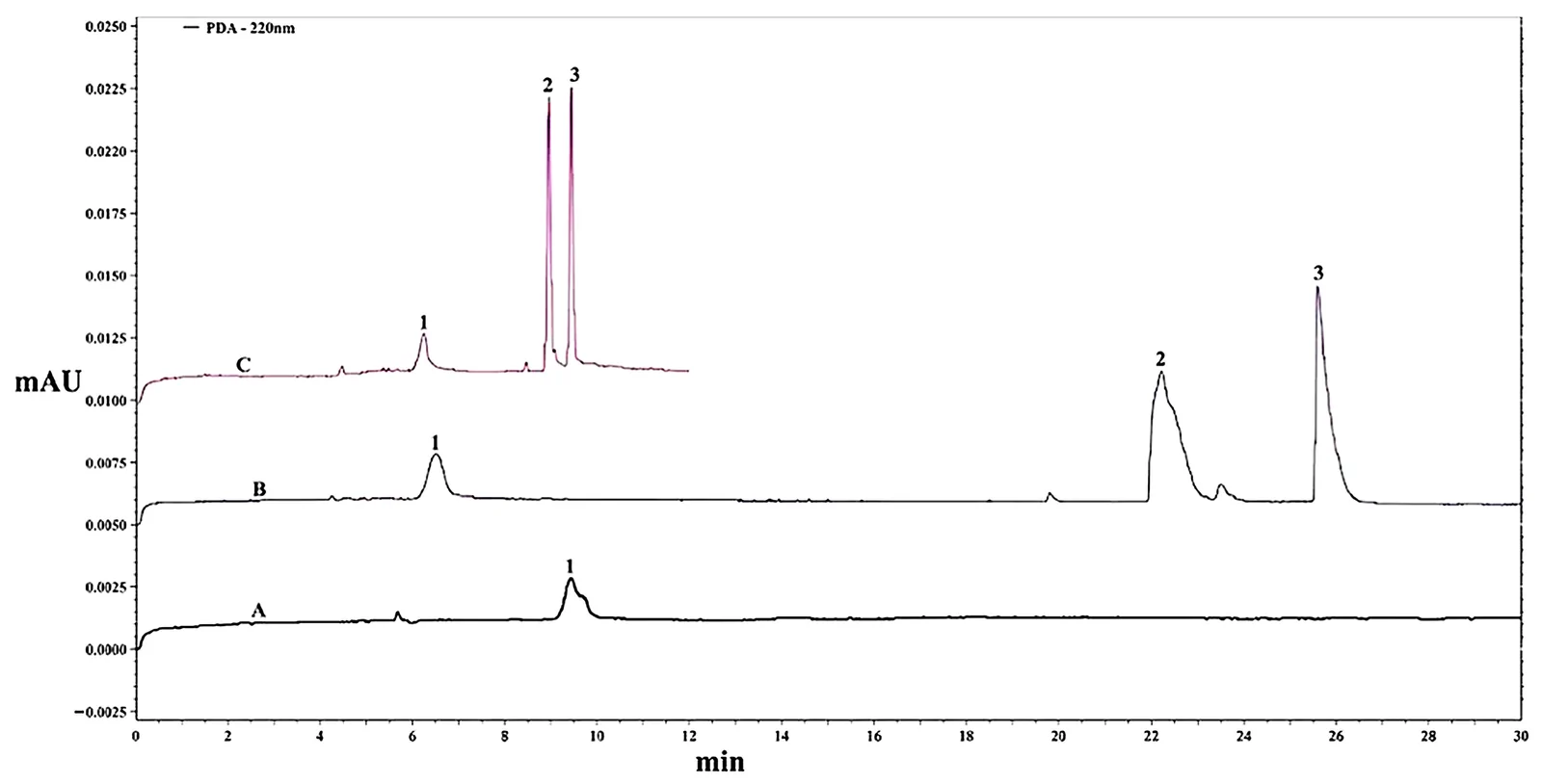
Initial separation trials under different conditions: (A) 10 mM phosphate, 40 mM SDS and 10% (*v*/*v*) acetonitrile, pH 8; (B) Condition A plus 0.5 mM Brij-35; (C) Condition A plus 5 mM Brij-35; applied voltage, 25 kV (detector at the cathode end); fused silica capillary, 50 cm (effective length: 40 cm) × 50 µm I.D.; UV detection at 220 nm. Peak identification: (1) doxycycline, (2) quinine, (3) chloroquine.

As shown in Figure 2 A, initial electrophoretic runs failed to achieve satisfactory separation of the target analytes. Among the three analytes, only doxycycline produced a detectable peak within 30 min, whereas chloroquine and quinine showed excessive retention. To modulate the EOF and optimize analyte partitioning between the aqueous phase and micellar pseudophase, key MEKC parameters were adjusted: pH (6.5–9.0), SDS concentration (20–70 mM), and organic modifier content (5–20%). Despite these systematic adjustments, doxycycline remained the only analyte migrating within an acceptable analysis window.

The observed separation behaviour may be explained by strong association of the antimalarials with the anionic SDS micelles. At the operational pH of 7.5, chloroquine and quinine are expected to remain substantially protonated and therefore carry a positive charge. Electrostatic attraction between these cationic species and the negatively charged sulfate headgroups of SDS may promote partitioning into the micellar pseudostationary phase, with hydrophobic interactions potentially providing an additional contribution. Consequently, strong analyte–micelle association could substantially increase effective retention and reduce the fraction of analyte migrating freely in the aqueous phase. Since SDS micelles exhibit electrophoretic migration toward the anode, strong association with the micelles would be expected to decrease the net migration velocity of the analytes. The resulting prolonged residence in the pseudostationary phase may also slow analyte exchange between the micellar and aqueous phases, which could contribute to peak broadening and reduced separation efficiency.

These results demonstrate that a simple SDS-MEKC system is insufficient for achieving simultaneous separation of all target analytes. The differences in charge state and micellar affinity necessitate either buffer modification, mixed-surfactant approaches, or alternative separation strategies to obtain acceptable resolution for the simultaneous analysis of these chemically diverse analytes.

#### 2.1.2. Effect of pH

Evaluation of the pH range of 6.5–9.0 identified pH 8.0 as optimal for the MEKC separation. At pH 8.0, the balance between the EOF, analyte ionization, and analyte–micelle interactions resulted in distinct effective mobilities and differential partitioning. These conditions produced symmetrical peak shapes, robust selectivity, and repeatable baseline resolution within an efficient analysis time. Therefore, pH 8.0 was selected for subsequent method optimization.

#### 2.1.3. Mixed Micellar System

To overcome the excessive analyte retention with SDS surfactant, a mixed-micelle system was explored by adding the non-ionic surfactant Brij-35 into the BGE. In the SDS-based system, strong electrostatic and hydrophobic interactions caused the basic antimalarial drugs to remain largely retained in the micellar phase, resulting in excessively long migration times. As shown in Figure 2 B, even adding as little as 0.5 mM Brij-35 to the conditions used in Figure 2 A produced a marked improvement in analyte behaviour. Under these modified conditions, both chloroquine and quinine, previously undetectable within 30 min, became detectable within this time window, demonstrating a clear reduction in micellar retention.

Increasing the Brij-35 concentration to 5 mM resulted in a further reduction in retention time. Compared with SDS and 0.5 mM Brij-35, the addition of 5 mM Brij-35 produced sharper and more symmetrical peaks, with reduced band broadening and substantially shorter analysis times. As illustrated in Figure 2 C, the 5 mM Brij-35 condition provided a favourable separation environment, in which the mixed SDS/Brij-35 micellar system moderated the strong interactions between the analytes and the SDS-rich phase [27]. This reduced excessive analyte retention and resulted in shorter migration times and improved overall separation efficiency.

#### 2.1.4. Optimization of Surfactant Concentrations

The concentrations of SDS and Brij-35 were optimized sequentially, while the phosphate concentration (10 mM), acetonitrile (ACN) content (10%, *v*/*v*), and pH (8) were maintained as constant throughout the optimization process. Initially, the Brij-35 concentration was fixed at 5 mM, while the SDS concentration was varied from 20 to 70 mM. Increasing the SDS concentration progressively improved the separation of the five antimalarial agents, as reflected by improved resolution and sharper peak profiles. The separation performance was optimal at 55 mM SDS. Increasing the SDS concentration further to 70 mM did not provide a further improvement in resolution but resulted in an increase in electrophoretic current. The higher current may promote Joule heating, which can adversely affect separation efficiency. Therefore, 55 mM SDS was selected for the subsequent optimization of Brij-35.

With the SDS concentration maintained at 55 mM, the Brij-35 concentration was subsequently evaluated from 5 to 30 mM, while the phosphate concentration, ACN content, and pH were maintained at 10 mM, 10% (*v*/*v*), and 8.0, respectively. Increasing the Brij-35 concentration altered the migration behaviour and peak profiles of the analytes. A concentration of 10 mM Brij-35 provided the most favourable overall separation, combining improved peak symmetry and resolution with a shorter analysis time. Further increases in Brij-35 concentration did not improve the separation and resulted in increased peak overlap. Accordingly, a BGE containing 55 mM SDS, 10 mM Brij-35, and 10 mM phosphate at pH 8.0 was selected for subsequent optimization of the organic modifier.

#### 2.1.5. Effect of Organic Modifier

Following optimization of the surfactant composition, with SDS and Brij-35 fixed at 55 and 10 mM, respectively, the effect of the organic modifier on the separation of the five antimalarial agents was investigated using the phosphate-based BGE employed during the preceding optimization steps. Increasing the organic modifier content improved the separation performance. However, precipitation was observed in the phosphate-containing BGE at higher organic modifier concentrations (>25% *v*/*v*), indicating insufficient BGE stability under these conditions. To overcome this limitation, the phosphate buffer was replaced with borate buffer. A 10 mM borate buffer provided improved BGE stability at higher organic modifier concentrations and was therefore selected for the subsequent optimization experiments.

Using the borate-based BGE, ACN and methanol (MeOH) were evaluated as organic modifiers. Compared with MeOH, ACN provided better overall separation performance, characterized by sharper and more symmetrical peaks, improved selectivity, and shorter migration times. ACN was therefore selected for further optimization of the organic modifier concentration.

The effect of ACN concentration was subsequently investigated over the range of 5–30% (*v*/*v*). Increasing the ACN content progressively altered the migration behaviour of the analytes and improved the overall separation. The most pronounced improvement was observed at 30% (*v*/*v*) ACN, at which the critical analyte pairs were adequately resolved and peak tailing was minimized. Accordingly, 30% (*v*/*v*) ACN was selected as the optimum organic modifier concentration, providing a favourable balance between resolution, peak shape, and analysis time.

#### 2.1.6. Effect of Surfactant Counterion on MEKC Separation

**Figure 3:**
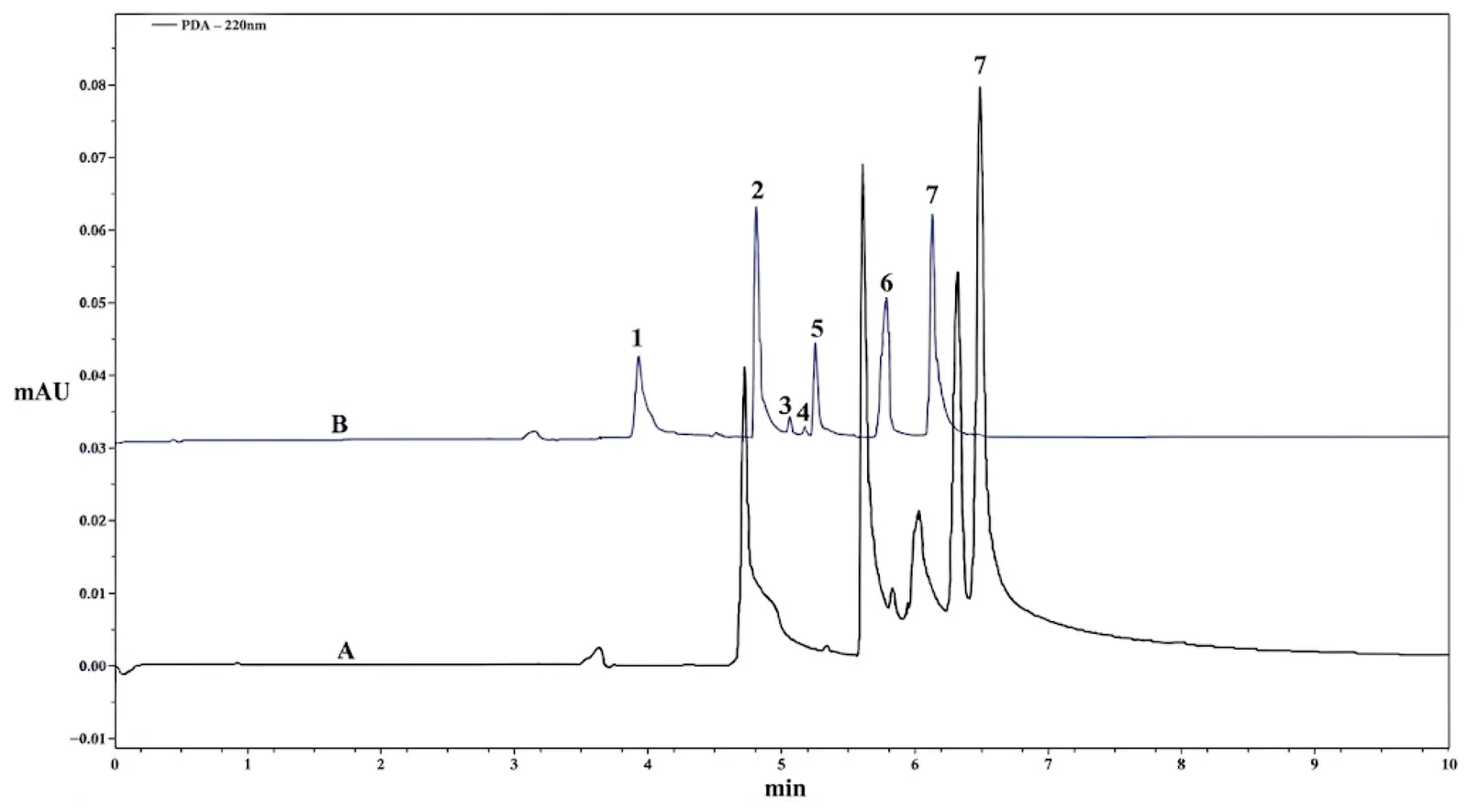
Comparison of SDS and LiDS surfactants under the optimized conditions; (A) 10 mM borate, 55 mM SDS, 10 mM Brij-35 and 30% (*v*/*v*) acetonitrile, pH 8; (B) 10 mM borate, 55 mM LiDS, 10 mM Brij-35 and 30% (*v*/*v*) acetonitrile, pH 8; applied voltage, 25 kV [detector at the cathode end]; fused silica capillary, 50 cm (effective length 40 cm) × 50 µm I.D.; UV detection at 220 nm; peak identification: (1) doxycycline, (2) quinine, (3) quinine impurity, (4) primaquine impurity, (5) primaquine, (6) mefloquine, (7) chloroquine.

Although the SDS/Brij-35 mixed-micelle system provided improved separation compared with SDS alone, the separation efficiency remained insufficient for reliable and rapid screening of the five antimalarial agents. Therefore, LiDS was investigated as an alternative anionic surfactant. As shown in Figure 3, replacing SDS with LiDS resulted in improved peak symmetry, enhanced baseline resolution, and overall better separation efficiency under the investigated conditions.

The improved performance of LiDS compared with SDS in the present MEKC system may be attributed to differences in their physicochemical and electrokinetic properties. Although both surfactants possess the same dodecyl sulfate headgroup and hydrophobic chain, their different counterions can influence micellar properties and the resulting separation behaviour. These differences can modify the micellar microenvironment and analyte partitioning between the aqueous phase and the micellar pseudostationary phase, thereby affecting migration and selectivity. The influence of the dodecyl sulfate counterion on MEKC separation has been demonstrated previously, with LiDS reported to provide higher separation efficiency and differences in retention compared with SDS, particularly for hydrophobic analytes in the presence of ACN [28]. Such effects may be particularly relevant for analytes with similar physicochemical properties, for which relatively small changes in micellar properties or analyte–micelle interactions can produce measurable differences in resolution. The improved peak symmetry and baseline resolution observed with LiDS therefore suggest that, under the present experimental conditions, LiDS provided a more favourable micellar environment for differential analyte separation than SDS.

**Figure 4:**
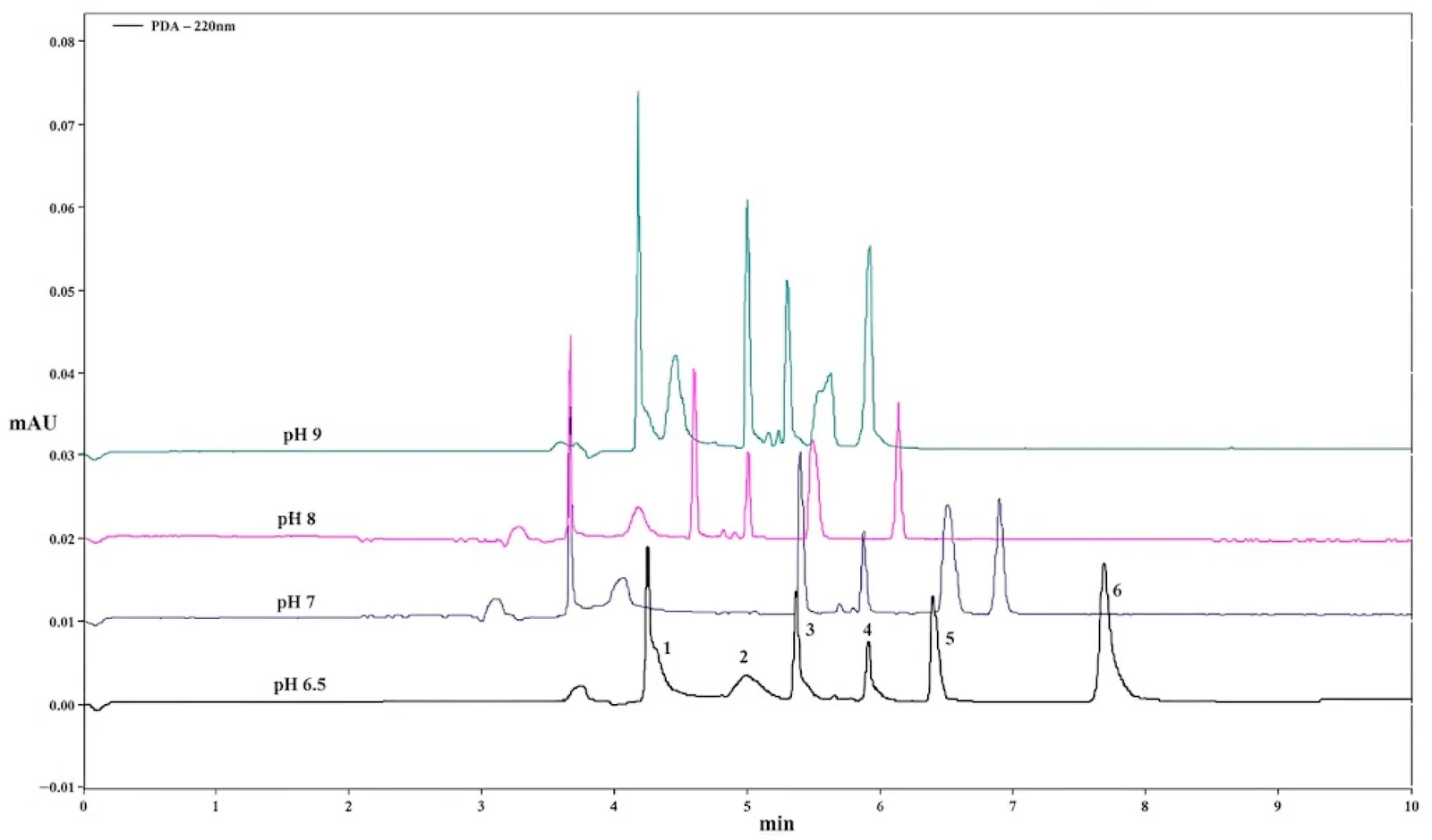
Representative electropherograms obtained at different pH values under the optimized conditions. Peak identification: (1) trimethoprim (internal standard), (2) doxycycline, (3) quinine, (4) primaquine, (5) mefloquine, (6) chloroquine.

To illustrate the influence of pH on separation performance, the effect of pH was re-evaluated under the optimized MEKC conditions. Representative electropherograms obtained at different pH values are shown in Figure 4. The results showed that pH 8.0 provided the best overall separation performance, with sharper, more symmetrical peaks and a shorter analysis time.

The final optimized MEKC method employed a BGE comprising 10 mM borate buffer, 55 mM LiDS, 10 mM Brij-35, and 30% (*v*/*v*) ACN, adjusted to pH 8.0.

**Figure 5:**
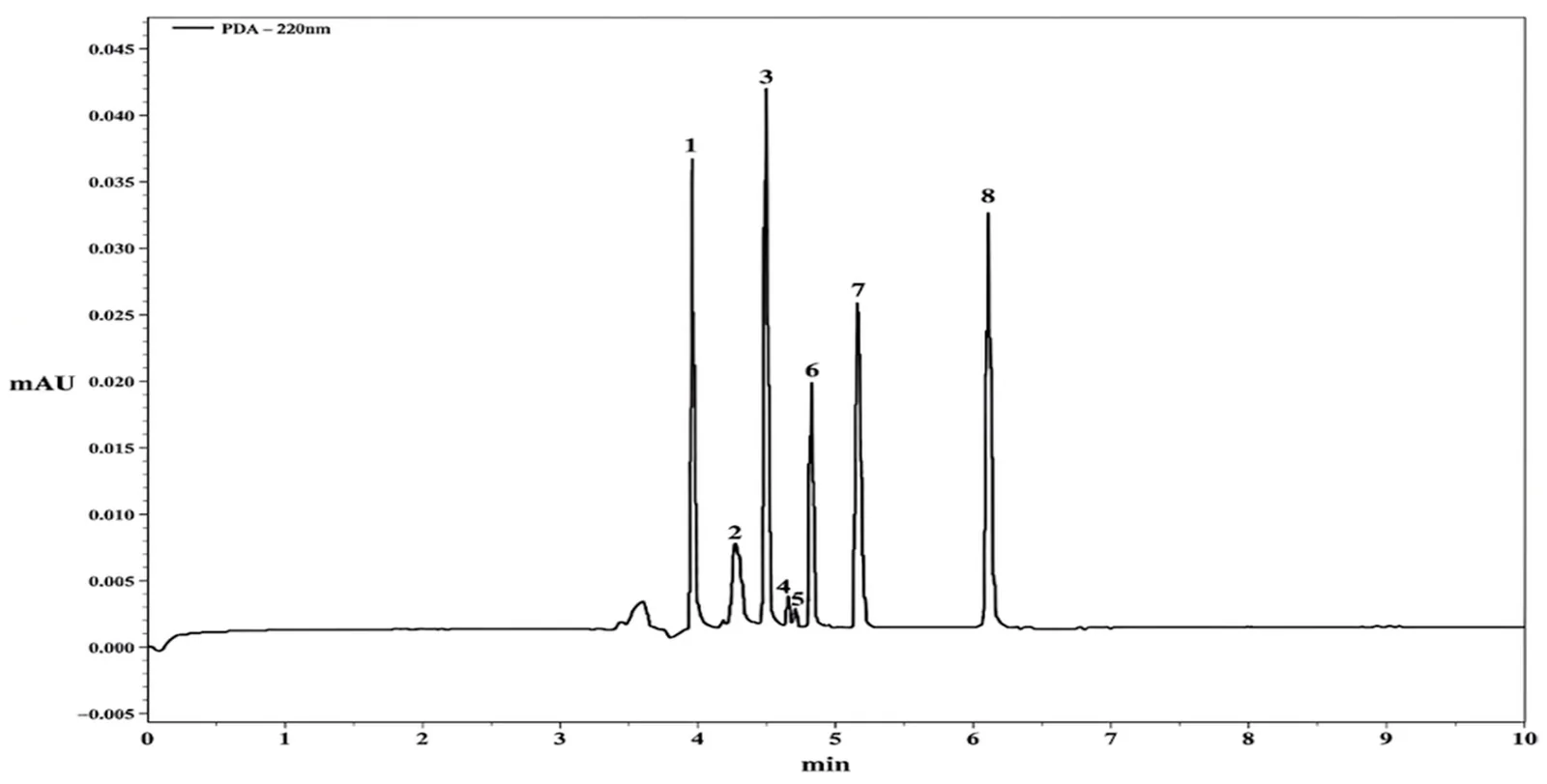
Representative electropherogram under the optimized conditions; peak identification: (1) trimethoprim (internal standard), (2) doxycycline, (3) quinine, (4) quinine impurity, (5) primaquine impurity, (6) primaquine, (7) mefloquine, (8) chloroquine.

Trimethoprim was used as the internal standard to compensate for variations in injection volume and other minor experimental fluctuations. Under the optimized conditions, complete baseline separation of the five antimalarial drugs with the IS was achieved within a short analysis time (Figure 5). The resulting method provided efficient and rapid separation, supporting its suitability for screening applications in low-resource settings.

**Figure 6:**
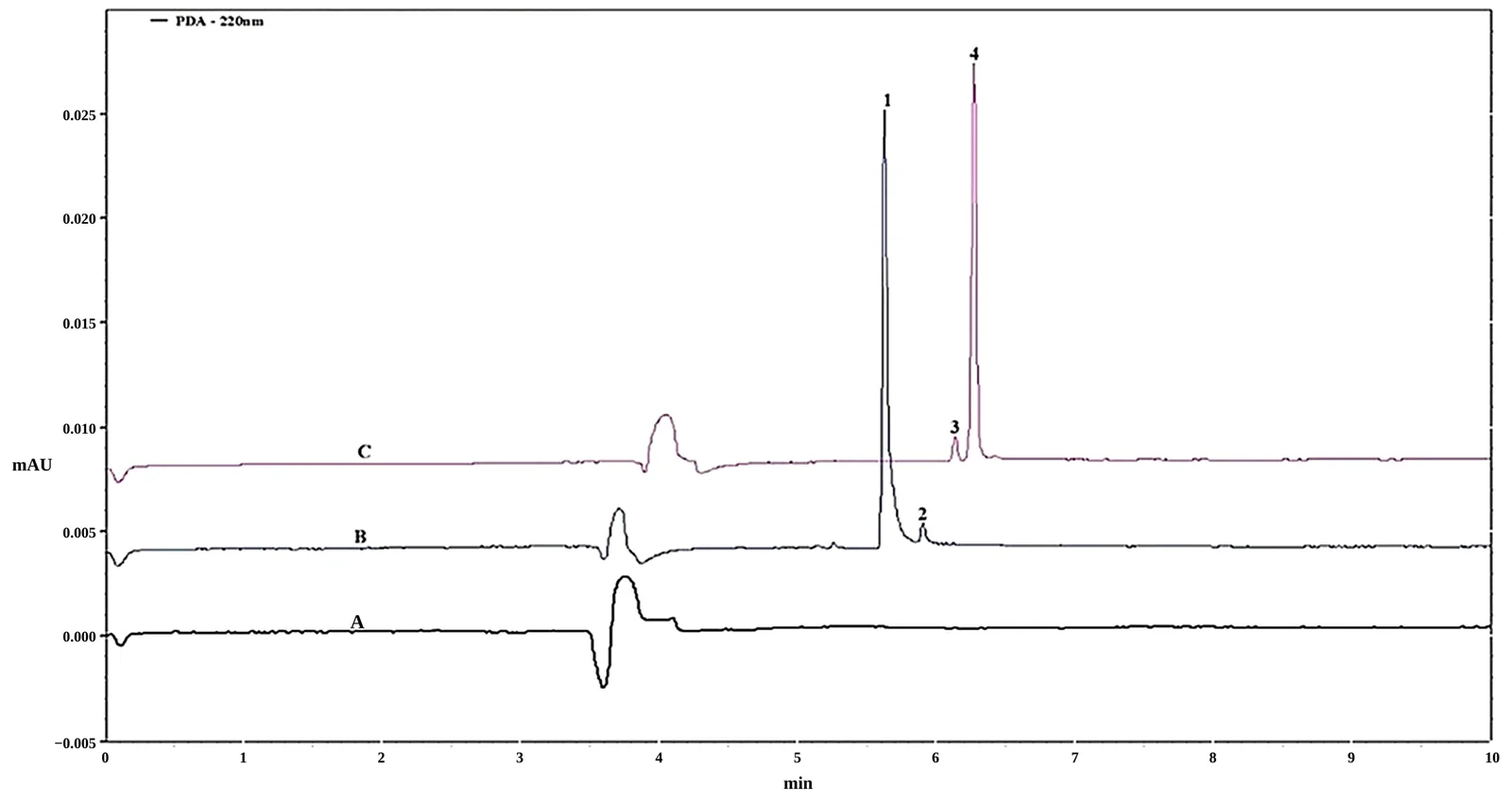
Overlaid electropherograms showing the source of impurity peaks under the optimized conditions; (A) solvent blank; (B) quinine standard; (C) primaquine standard. Peak identification: (1) quinine, (2) quinine impurity, (3) primaquine impurity, (4) primaquine.

As shown in Figure 5, the electropherograms consistently displayed two additional minor peaks eluting between the quinine and primaquine peaks (peaks 4 and 5). Because these signals were consistently observed, additional experiments were conducted to investigate their origin (Figure 6). Injection of the solvent blank produced a stable baseline without detectable signals at the corresponding migration times, indicating that the additional peaks were unlikely to originate from the BGE, solvent, or other system-related sources.

Each reference standard was subsequently analysed individually to determine whether the additional signals were associated with specific analytes. The minor peaks were observed only in the quinine and primaquine standard solutions. Injection of the quinine standard produced the main quinine peak accompanied by a smaller peak migrating immediately after it, whereas the primaquine standard produced a minor peak eluting immediately before the main primaquine peak. The consistent appearance of these signals in the individual standard solutions indicates that they are associated with the respective reference materials and may correspond to trace impurities and/or minor degradation products. However, their exact chemical identities could not be confirmed without further characterization, such as mass spectrometric analysis.

Importantly, the optimized mixed-micellar system provided baseline resolution of these minor signals from the five target analytes and the internal standard, trimethoprim. Hence, these additional peaks did not interfere with analyte identification or quantification under the optimized conditions. Their consistent resolutions throughout the analyses further support the selectivity and suitability of the developed MEKC method for the intended screening application.

### 2.2. Method Validation

The optimized method was validated in terms of robustness, selectivity, repeatability, sensitivity, and linearity.

#### 2.2.1. Robustness

The resolution (Rs) between quinine and its associated impurity peak, and between primaquine and its associated impurity peak, was selected as a critical performance criterion of the method. Adequate resolution of these peak pairs is essential for reliable identification and quantification of the respective analytes in the presence of structurally related impurities. Robustness was assessed by evaluating changes in Rs following small, deliberate variations in key electrophoretic parameters.

The robustness assessment was performed using a two-level fractional factorial experimental design implemented in R software. Four electrophoretic parameters with potential effects on migration behaviour, selectivity, resolution, and peak shape were investigated: LiDS concentration, Brij-35 concentration, ACN content, and pH. Each factor was varied symmetrically around its nominal value, with the selected levels representing realistic deviations that may occur during routine operation. The investigated factor levels are summarized in Table 1.

**Figure 7:**
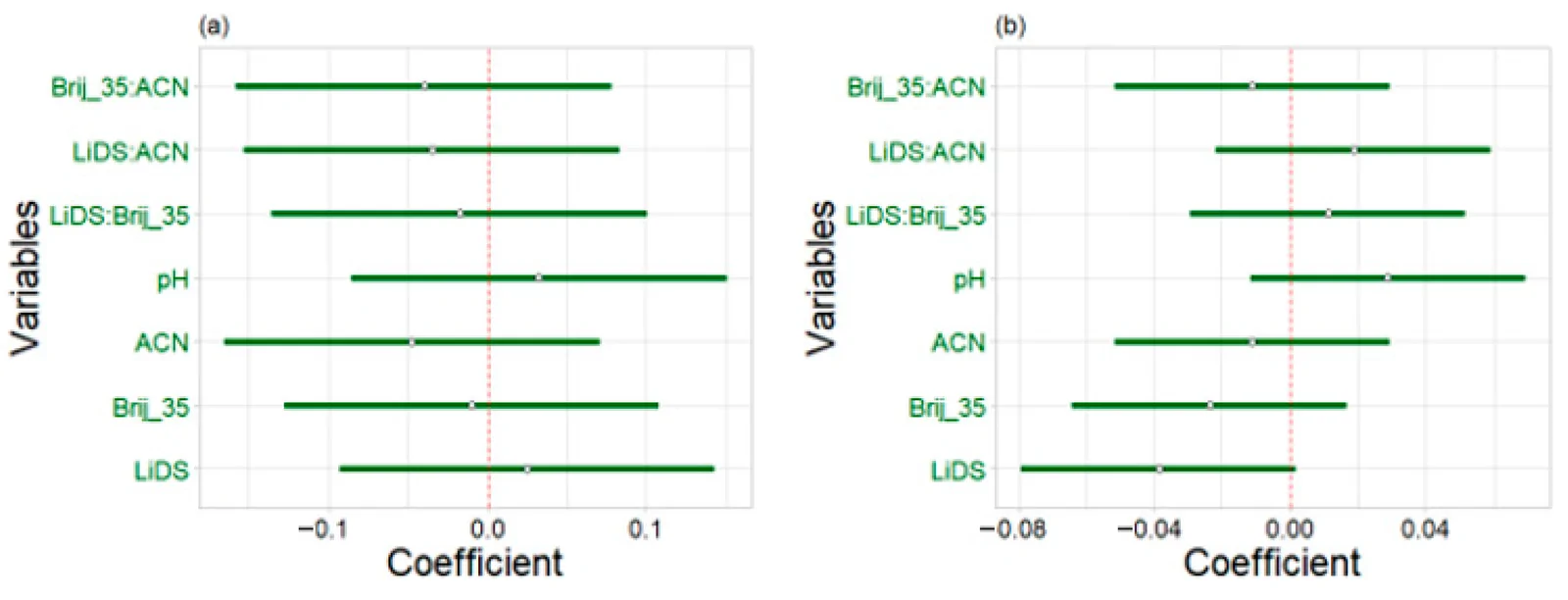
Regression coefficient plots for (**a**) resolution between quinine and its impurity peak and (**b**) resolution between primaquine and its impurity peak. The horizontal lines represent the 95% confidence interval.

The effects of the selected parameters were evaluated using regression analysis in R, with the resolution of the quinine/impurity (Rs_1_) and primaquine/impurity (Rs_2_) peak pairs used as the response variables. No statistically significant effects were detected among the main factors or two-factor interactions included in the model. As shown in Figure 7, the 95% confidence intervals for all estimated effects crossed zero, indicating that the observed variations in resolution were not attributable to any statistically significant systematic effect.

The resolution of both critical peak pairs remained above the generally accepted threshold of 1.5 under all experimental conditions. Although minor fluctuations in resolution were observed across runs, these did not compromise the required electrophoretic separation of quinine and primaquine from their respective impurities. Overall, the robustness study demonstrated that the method maintained adequate separation of the critical analyte–impurity pairs despite small, deliberate variations in the selected electrophoretic parameters.

#### 2.2.2. Selectivity

**Figure 8:**
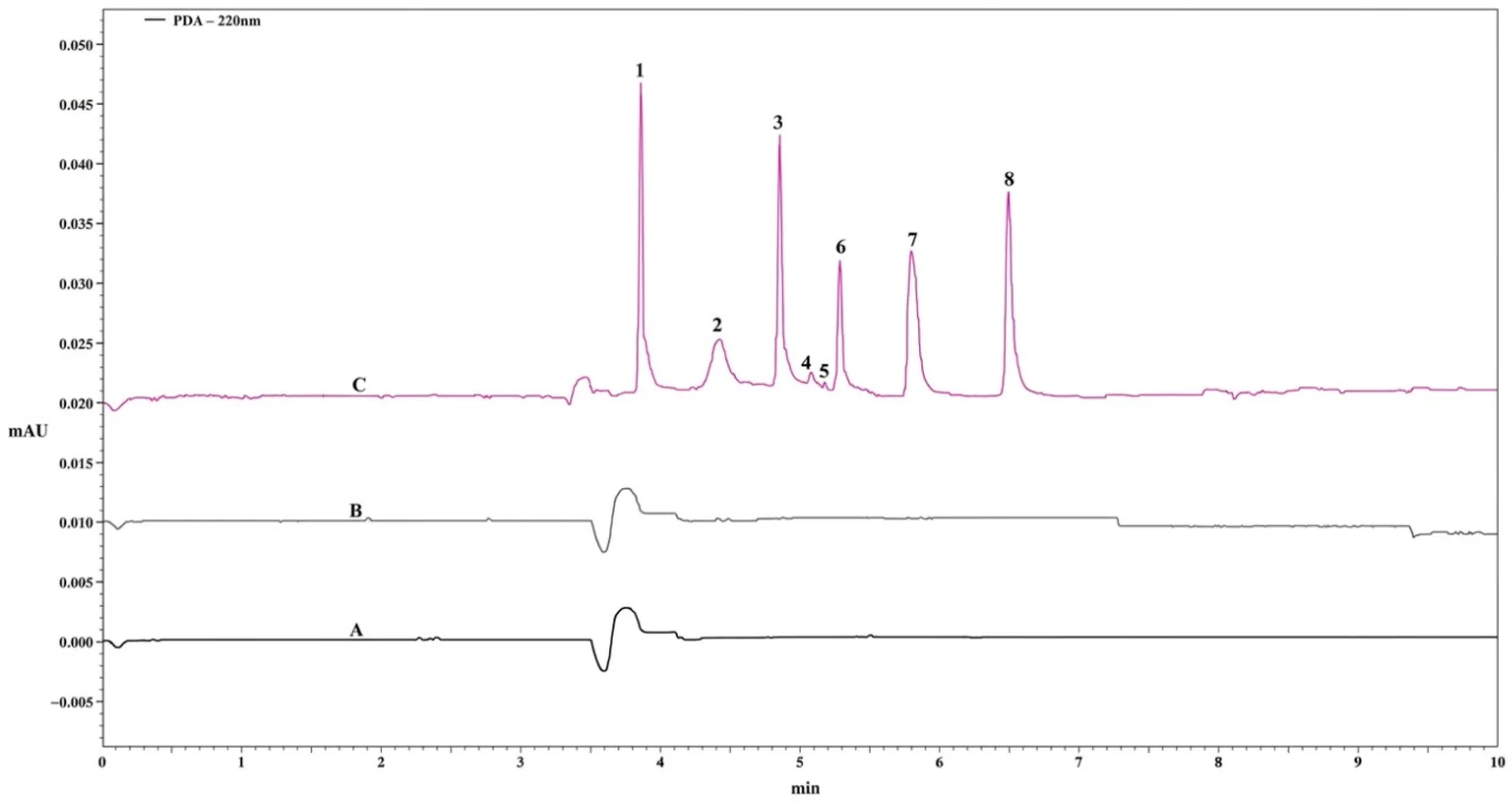
Overlaid electropherograms demonstrating the selectivity of the developed method under the optimized conditions: (A) solvent blank solution, (B) excipient solution, (C) mixed antimalarial standards and the internal standard. Peak identification: (1) trimethoprim (internal standard), (2) doxycycline, (3) quinine, (4) quinine impurity, (5) primaquine impurity, (6) primaquine, (7) mefloquine, (8) chloroquine.

Selectivity was evaluated by comparing electrophoretic profiles obtained from the solvent blank, placebo, internal standard, and antimalarial drug standards. The placebo contained common tablet excipients, including magnesium stearate, talc, croscarmellose sodium, mannitol, and lactose monohydrate. Electropherograms were examined at the selected detection wavelength to determine whether any components originating from the BGE or formulation excipients produced signals that overlapped with those of the target analytes. As shown in the overlaid electropherograms in Figure 8, no peaks from the blank or placebo appeared at the migration times of the antimalarial drugs or the internal standard. These results confirm the absence of matrix-related interference and demonstrate that the method provides adequate selectivity for the determination of the antimalarial drugs in pharmaceutical formulations.

#### 2.2.3. Precision

Precision was evaluated in terms of repeatability by determining the %RSD of the corrected peak areas and migration times for each antimalarial drug. Migration-time repeatability was excellent, with %RSD values below 2% for all analytes. The peak-area precision was also very good, with %RSD values ranging from 1.4% to 3.1%. Detailed repeatability data are presented in Table 2.

#### 2.2.4. Linearity

Linearity was assessed by constructing calibration curves for each analyte, with peak area ratios corrected for migration time variation and normalized to the internal standard plotted against the corresponding analyte concentration. Doxycycline was evaluated over the concentration range of 0.2–0.6 mg/mL, while chloroquine, quinine, primaquine, and mefloquine were evaluated over the range of 0.1–0.3 mg/mL. All analytes showed good linearity over the evaluated concentration ranges, with coefficients of determination (r^2^) greater than 0.99. Residual analysis showed a random distribution of residuals around zero without any systematic pattern, further supporting the suitability of the linear model. These results confirmed the linear response of the method over the concentration ranges investigated.

#### 2.2.5. Limit of Detection and Quantitation

The limits of detection (LOD) and quantitation (LOQ) were determined using the signal-to-noise (S/N) ratio approach. An S/N ratio of approximately 3:1 was used to define the LOD, whereas an S/N ratio of approximately 10:1 was used to define the LOQ. Serial dilutions of the analytes were analysed under the optimized conditions, and the resulting signals were evaluated relative to the baseline noise. The LOD values ranged from 0.0009 to 0.02 mg/mL, while the LOQ values ranged from 0.0030 to 0.07 mg/mL. The LOD and LOQ values obtained for each analyte are summarized in Table 2.

Although some previously reported CE methods achieved lower detection limits for individual antimalarial drugs or selected drug combinations, they were generally developed for a more limited range of analytes. In contrast, the proposed mixed-micelle CE method enables the simultaneous determination of five antimalarial drugs within 7 min. The achieved detection limits are sufficient for pharmaceutical quality-screening applications, while the method provides broader analyte coverage, rapid analysis, and higher analytical throughput, potentially reducing the overall cost per analysis.

### 2.3. Application to Commercial Products

To evaluate the applicability of the developed MEKC-UV method to pharmaceutical formulations, commercially available antimalarial products obtained from the Ethiopian market were analyzed. The investigated products included doxycycline hyclate capsules, chloroquine phosphate tablets, and primaquine phosphate tablets. Using the optimized sample preparation and separation conditions, the declared active pharmaceutical ingredients (APIs) were successfully identified and quantified.

**Figure 9:**
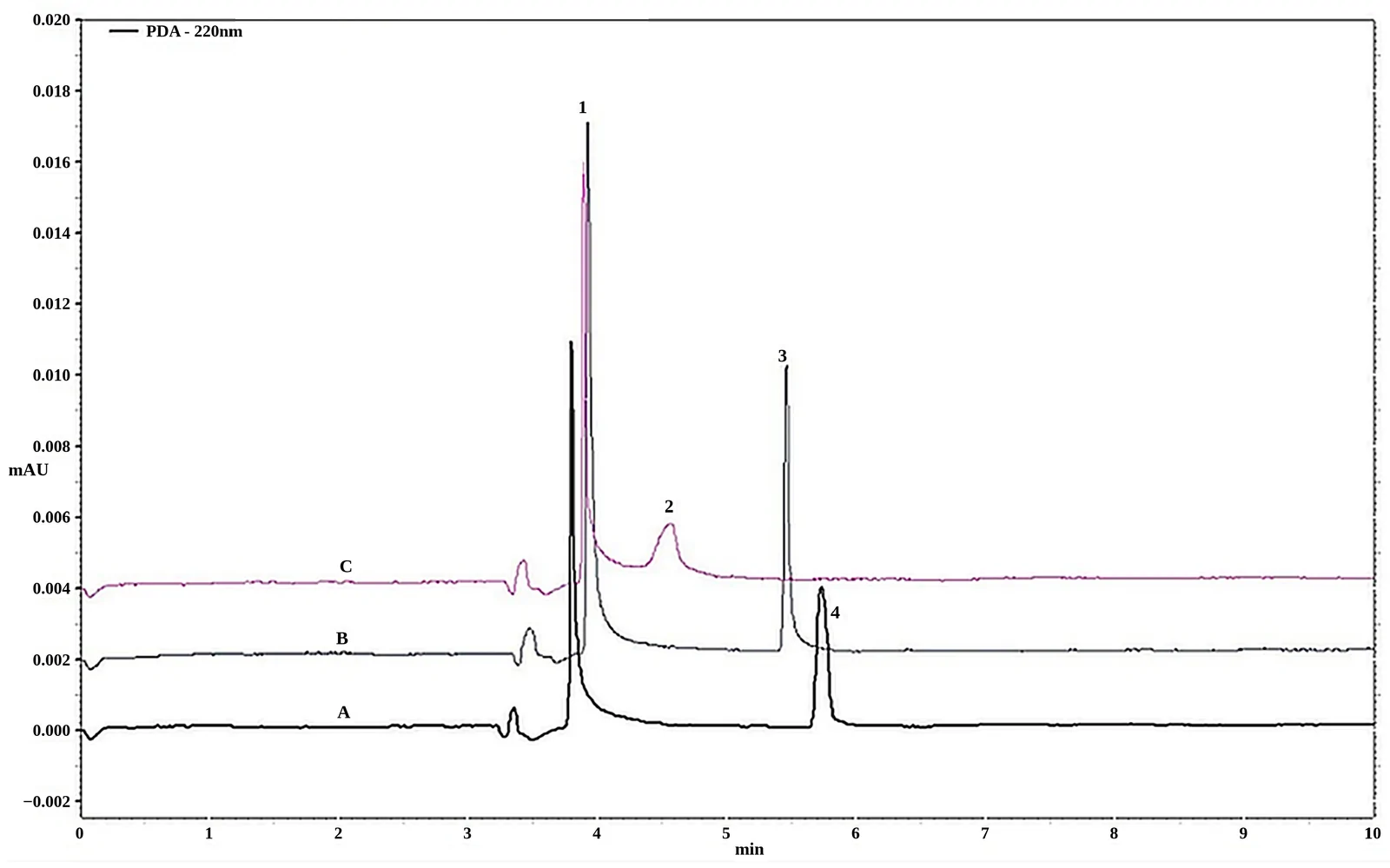
Representative electropherograms of commercial samples: (A) chloroquine phosphate tablets, (B) primaquine phosphate tablets, and (C) doxycycline hyclate capsules. Peak identification: (1) trimethoprim (internal standard, IS), (2) doxycycline, (3) primaquine, and (4) chloroquine.

Representative electropherograms are presented in Figure 9, and the assay results are summarized in Table 3. All formulations produced well-resolved analyte peaks with migration times consistent with those of the corresponding reference standards, confirming the identity of the labeled APIs. The assay results were within the USP acceptance ranges [29] for the respective products and therefore provide preliminary evidence of the applicability of the proposed method to commercial pharmaceutical formulations. However, because accuracy studies involving recovery assessment and evaluation of potential matrix effects during sample preparation were not included in the present work, the analytical performance of the method in commercial samples should be considered preliminary. Further validation studies are required before routine quality-control implementation can be recommended.

## 3. Experiment

### 3.1. Chemicals and Reagents

The reference standards of chloroquine sulfate, quinine sulfate, primaquine diphosphate, mefloquine hydrochloride, doxycycline hyclate, and trimethoprim were obtained from the European Directorate for the Quality of Medicines (Strasbourg, France). ACN and methanol (HPLC grade), as well as SDS and LiDS, were supplied by Acros Organics (Geel, Belgium). Brij-35 was purchased from Sigma-Aldrich (St. Louis, MO, USA). Sodium tetraborate decahydrate and disodium hydrogen phosphate were obtained from Sigma-Aldrich and Merck KGaA (Darmstadt, Germany), respectively.

The excipients magnesium stearate, talc, croscarmellose sodium, mannitol, and lactose monohydrate were provided by Fagron (Waregem, Belgium). Sodium hydroxide (NaOH) was purchased from VWR (Leuven, Belgium), while hydrochloric acid (HCl) and phosphoric acid (H_3_PO_4_) were obtained from Fisher Scientific (Loughborough, UK). A 0.45 µm membrane filter (MACHEREY-NAGEL GmbH & Co. KG, Düren, Germany) was used for solution filtration. Ultrapure water was prepared using a Milli-Q purification system (Millipore, Bedford, MA, USA).

### 3.2. Instrumentation and CE Parameters

The analyses were performed on a Beckman P/ACE^TM^ MDQ system (Brea, CA, USA) equipped with UV detection and a temperature control unit. Separations were carried out in a fused-silica capillary (50 cm total length, 40 cm effective length, 50 µm I.D.) from BGB (Harderwijk, The Netherlands). The capillary was maintained at 25 °C by immersion in a circulating liquid coolant. UV detection was set at 220 nm, with the cathode positioned at the detection end. Samples were introduced hydrodynamically at 3.5 kPa for 5 s, and electrophoretic separations were conducted at 25 kV with the anode at the injection side.

New capillaries were conditioned by sequential rinsing with 1 M NaOH (25 min), water (5 min), and the BGE (5 min). Prior to each run, the capillary was flushed with 0.1 M NaOH (2 min), water (2 min), and BGE (3 min). All rinsing steps were performed at 138 kPa. Instrument control and data processing were carried out using 32 Karat software, version B.04.02 (Beckman, Brea, CA, USA). To minimize the risk of capillary blockage, the capillary was rinsed at the end of each day with 1 M NaOH (10 min) followed by Milli-Q water (10 min).

### 3.3. Preparation of Standard Solutions

Standard solutions were prepared using an 80:20 (*v*/*v*) water–methanol mixture, whereas Milli-Q water was used for all other solutions. Fresh standard solutions of the antimalarial drugs (chloroquine, quinine, primaquine, and mefloquine) and internal standard (trimethoprim) were prepared at 0.2 mg/mL, and doxycycline at 0.4 mg/mL. Following analysis, the remaining solutions were transferred to tightly sealed amber containers and stored at 2–8 °C to ensure stability and light protection.

### 3.4. Preparation of Sample Solutions

Ten tablets or capsules from each product were accurately weighed. Tablets were finely powdered, whereas capsules were opened and their contents mixed. An amount of powder corresponding to a nominal concentration of 0.2 mg/mL chloroquine, 0.2 mg/mL primaquine, and 0.4 mg/mL doxycycline was accurately weighed and dissolved in an 80:20 (*v*/*v*) water–methanol mixture. Trimethoprim was added as the internal standard to make a final concentration of 0.2 mg/mL. The resulting solutions were thoroughly mixed and filtered through a 0.45 μm membrane filter prior to analysis.

### 3.5. Preparation of BGE

The BGE was prepared by dissolving the appropriate amount of sodium tetraborate salt in Milli-Q water to obtain a final borate concentration of 10 mM. LiDS and Brij-35 were subsequently added to obtain final concentrations of 55 and 10 mM, respectively, followed by the addition of ACN to a final concentration of 30% (*v*/*v*). The apparent pH of the resulting BGE was adjusted to 8.0 using 1 M HCl, and the solution was brought to the final volume with Milli-Q water and thoroughly mixed. Prior to use, the BGE was filtered using a 0.45 µm membrane filter.

### 3.6. Method Development and Optimization

Method development was performed to establish an efficient MEKC method for the simultaneous separation of the five antimalarial drugs and the internal standard. Given the diverse physicochemical and ionization characteristics of the selected analytes, MEKC was selected as the separation mode. The major electrophoretic separation parameters were systematically investigated to identify conditions providing adequate resolution, desirable peak shape, and short analysis time. The compositions of buffer salt, surfactant type and concentration, organic modifier type and proportion, and pH were evaluated and optimized.

#### 3.6.1. Selection of Buffer Salt

Borate and phosphate buffers were initially evaluated as the aqueous buffer components of the BGE. Different buffer concentrations and pH conditions were investigated to assess their effects on analyte migration, peak resolution, and current generation. Phosphate buffer was used during the initial surfactant optimization, while borate buffer was subsequently selected because of its greater stability in the presence of the higher concentration of organic modifier.

#### 3.6.2. Evaluation of Surfactants

SDS was initially investigated as the anionic surfactant, followed by the addition of the non-ionic surfactant Brij-35 to form a mixed-micellar system and improve separation performance. The concentrations of SDS and Brij-35 were subsequently optimized based on analyte migration, peak shape, resolution, and efficiency. The SDS/Brij-35 system was first evaluated, followed by assessment of the LiDS/Brij-35 system with an alternative anionic surfactant.

#### 3.6.3. Evaluation of Organic Modifiers

ACN and methanol were investigated as organic modifiers of the BGE. Their proportions were evaluated to determine their effects on the viscosity and electroosmotic flow of the separation medium, as well as on analyte–micelle interactions and selectivity. The organic modifier providing the most favourable overall separation characteristics was selected for further optimization.

#### 3.6.4. Optimization of pH

The pH of the BGE was systematically investigated because it directly affected the EOF, the ionization state of the analytes, and their interaction with the micellar phase. An appropriate pH was selected based on its ability to provide adequate and repeatable separation of the five antimalarial medicines while maintaining acceptable migration times and peak characteristics.

#### 3.6.5. Selection of Internal Standard

Trimethoprim was incorporated as the internal standard to compensate for variations in injection volume and instrumental response during analysis. Its suitability was assessed based on its migration behaviour, resolution from the analyte peaks, and absence of interference with the target compounds.

### 3.7. Method Validation

Following optimization, the developed method was validated with respect to robustness, linearity, repeatability, selectivity, LOD, and LOQ, in accordance with ICH Q2(R2) guidelines [30].

Robustness was evaluated using a two-level fractional factorial experimental design with three center points. As shown in Table 1, four critical electrophoretic parameters were investigated: LiDS concentration (LiDS), Brij-35 concentration (Brij-35), acetonitrile content (ACN), and pH. The analytical responses were the resolution values for the quinine-related impurity peak pair (Rs_1_) and the primaquine-related impurity peak pair (Rs_2_). These responses were used to assess the effects of small deliberate variations in the parameters investigated on the separation performance of the optimized method.

The total number of experimental runs was calculated according to:

$$N=2^{k-p\;}+nc$$

where (*N*) is the total number of experimental runs, (*k*) is the number of investigated factors, (*p*) represents the degree of fractionation, and (*nc*) is the number of center-point experiments. In the present study, four factors (*k* = 4) were investigated using a half fractional factorial design (*p* = 1), with three center points (*nc* = 3).

$$N=2^{4-1\;}+3=11$$

Accordingly, the experimental design comprised eight factorial runs and three center-point runs. The design matrix was generated and randomized using the FrF2 package in R software, version 4.6.1 (R Foundation for Statistical Computing, Vienna, Austria, 2026). The effects of the individual main factors and selected two-factor interactions on the analytical response variables, Rs_1_ and Rs_2_, were evaluated using multiple linear regression.

The regression coefficients and their corresponding 95% confidence intervals were graphically represented using the ggplot2 package in R. The resulting coefficient plots were used to visualize the estimated effects of the investigated factors and interactions on the separation performance. An effect was considered statistically significant at the 5% significance level when its 95% confidence interval did not include zero, whereas effects with confidence intervals including zero were considered not statistically significant. This analysis was used to assess the sensitivity of the separation to small variations in the electrophoretic parameters investigated and to evaluate the robustness of the optimized method.

Selectivity was evaluated using a placebo mixture containing magnesium stearate, talc, croscarmellose sodium, mannitol, and lactose monohydrate. These excipients were selected as representative excipients frequently used in tablet and capsule formulations. The placebo mixture was analyzed under the optimized MEKC conditions to assess potential interference from formulation excipients at the migration times of the target analytes. Method selectivity was further evaluated by assessing the ability of the optimized MEKC conditions to separate the target antimalarial drugs from one another and from impurity peaks present in the quinine and primaquine reference standards.

Precision was determined in terms of repeatability by analyzing six replicate injections of standard solutions prepared at the nominal assay concentration (100%) under the same operating conditions on the same day.

Linearity was assessed over concentration ranges of 0.2–0.6 mg/mL for doxycycline and 0.1–0.3 mg/mL for chloroquine, quinine, primaquine, and mefloquine. Calibration curves were constructed using five concentration levels corresponding to 50%, 75%, 100%, 125%, and 150% of the target assay concentration, with each level analyzed in triplicate. Linear regression analysis was performed by plotting the analyte-to-internal-standard (trimethoprim) peak-area ratio against analyte concentration. The suitability of the calibration model was evaluated based on the coefficient of determination (R^2^), intercept, and residual plots.

Sensitivity was assessed by determining the LOD and LOQ. Serial dilutions of each analyte were prepared and analyzed under the optimized MEKC conditions. Baseline noise was measured as the peak-to-peak noise in a signal-free region of the electropherogram adjacent to the analyte peak. The LOD and LOQ were defined as the analyte concentrations corresponding to S/N ratios of approximately 3:1 and 10:1 respectively.

## 4. Conclusions

This study developed and systematically optimized an MEKC method for the simultaneous screening of five antimalarial drugs: chloroquine, doxycycline, quinine, primaquine, and mefloquine. The optimization demonstrated the importance of surfactant composition in controlling the migration and separation of these analytes. Initial SDS-based MEKC conditions resulted in excessive retention of the basic analytes. The incorporation of Brij-35 helped to moderate analyte–micelle interactions and improved the overall separation behaviour. Further optimization using LiDS resulted in improved peak symmetry, migration behaviour, and selectivity compared with the SDS-based system.

The optimized BGE, consisting of 10 mM borate buffer, 55 mM LiDS, 10 mM Brij-35, and 30% acetonitrile at pH 8.0, provided efficient and repeatable separation. Under these conditions, complete baseline separation of the five antimalarial drugs and the internal standard was achieved within a short analysis time. The method also consistently resolved minor impurity peaks observed in quinine and primaquine standards, demonstrating its ability to distinguish additional components without compromising the separation of the target analytes.

Analytical validation demonstrated good robustness, repeatability, and linearity (r^2^ > 0.99), and detection limits suitable for screening applications. However, an investigation on the recovery and matrix effect was not included in the current study. Overall, the optimized MEKC method provides a rapid, reliable, and affordable approach for antimalarial medicine-quality screening and shows potential as a practical analytical tool for medicine-quality surveillance, particularly in resource-limited settings. Finally, the method was applied to the analysis of commercially available antimalarial products. All analyzed products complied with the acceptance criteria specified in the relevant USP monographs. These findings confirm the potential applicability of the developed MEKC method for the reliable identification and quantification of antimalarial drugs in pharmaceutical dosage forms.

## Figures and Tables

**Table 1:** Electrophoretic parameters and levels used in the robustness assessment.

Parameter	Low Value (−)	Central Value (0)	High Value (+)
LiDS (mM)	50	55	60
Brij-35 (mM)	8	10	12
ACN (*v*/*v*, %)	27.5	30	32.5
pH	7.8	8.0	8.2

**Table 2:** Results of validation study.

Parameters	Doxycycline	Quinine	Primaquine	Mefloquine	Chloroquine
RSD (%) migration time	1.1	0.8	0.7	0.5	0.5
RSD (%) peak area	2.6	2.4	1.4	3.1	2.0
Linearity range (mg/mL)	0.2–0.6	0.1–0.3	0.1–0.3	0.1–0.3	0.1–0.3
r^2^	0.9987	0.9925	0.9923	0.9931	0.9928
LOD (mg/mL)	0.0207	0.0009	0.0035	0.0015	0.0012
LOQ (mg/mL)	0.069	0.0030	0.0118	0.0051	0.0040

**Table 3:** Assay results of commercial samples.

Analyzed Sample	Country of Origin	Assay (% Label Claim) (%RSD, n = 3 Injections)	USP Acceptance Range (%)
Doxycycline hyclate 100 mg capsule	India	91.6 (2.9)	90.0–120.0
Chloroquine phosphate 15 mg tablet	India	93.4 (2.6)	93.0–107.0
Primaquine 250 mg tablet	India	95.2 (2.2)	93.0–107.0

## Data Availability

All data supporting the findings of this study are available from the authors upon reasonable request.

## References

[1] World Health Organization (2024). World Malaria Report 2024.

[2] Zhang S.X., Yang G.B., Yang J., Wei F.N., Lv S., Duan L., Tian L.-G., Chen M.-X., Liu Q., Lu Y. (2024). Global, regional, and national burden of malaria, 1990–2021: Findings from the global burden of disease study 2021. Decod. Infect. Transm..

[3] World Health Organization (2024). Malaria Fact Sheet.

[4] Popoola O.O., Madhur G., Mehrim M.M., Omondi M.O., Owusu-Mensah P., Mamtani S.A., Etukakpan A.U. (2022). A literature review on the global burden and impact of substandard and falsified medicine. Ann. Public Health Issues.

[5] Ozawa S., Evans D.R., Bessias S., Haynie D.G., Yemeke T.T., Laing S.K., Herrington J.E. (2018). Prevalence and estimated economic burden of substandard and falsified medicines in low- and middle-income countries: A systematic review and meta-analysis. JAMA Netw. Open.

[6] Evans D.R., Higgins C.R., Laing S.K., Awor P., Ozawa S. (2019). Poor-quality antimalarials further health inequities in Uganda. Health Policy Plan..

[7] Bui V., Higgins C.R., Laing S., Ozawa S. (2022). Assessing the impact of substandard and falsified antimalarials in Benin. Am. J. Trop. Med. Hyg..

[8] Ozawa S., Evans D.R., Higgins C.R., Laing S.K., Awor P. (2019). Development of an agent-based model to assess the impact of substandard and falsified anti-malarials: Uganda case study. Malar. J..

[9] Rosenthal P.J., Asua V., Bailey J.A., Conrad M.D., Ishengoma D.S., Kamya M.R., Rasmussen C., Tadesse F.G., Uwimana A., Fidock D.A. (2024). The emergence of artemisinin partial resistance in Africa: How do we respond?. Lancet Infect. Dis..

[10] Rosenthal P.J., Asua V., Conrad M.D. (2024). Emergence, transmission dynamics and mechanisms of artemisinin partial resistance in malaria parasites in Africa. Nat. Rev. Microbiol..

[11] Beargie S.M., Higgins C.R., Evans D.R., Laing S.K., Erim D., Ozawa S. (2019). The economic impact of substandard and falsified antimalarial medications in Nigeria. PLoS ONE.

[12] World Health Organization (2020). A Study on the Public Health and Socioeconomic Impact of Substandard and Falsified Medical Products.

[13] Chabalenge B., Sahota T., Ermolina I., Tanna S. (2025). Substandard and falsified medicines in Africa: Healthcare systems challenges, supply chain issues, regulatory challenges and strategies to increase access to quality medicines. Front. Pharmacol..

[14] Petersen A., Held N., Heide L., Difäm-EPN Minilab Survey Group (2017). Surveillance for falsified and substandard medicines in Africa and Asia by local organizations using the low-cost GPHF Minilab. PLoS ONE.

[15] Gnegel G., Häfele-Abah C., Neci R., Heide L., Difäm-EPN Minilab Network (2022). Surveillance for substandard and falsified medicines by local faith-based organizations in 13 low- and middle-income countries using the GPHF Minilab. Sci. Rep..

[16] Amin N.C., Blanchin M.D., Aké M., Fabre H. (2013). Capillary electrophoresis methods for the analysis of antimalarials. Part II. Achiral separative methods. J. Chromatogr. A.

[17] Amin N.C., Blanchin M.D., Aké M., Fabre H. (2012). Capillary electrophoresis methods for the analysis of antimalarials. Part I. Chiral separation methods. J. Chromatogr. A.

[18] Amin N.C., Blanchin M.D., Aké M., Montels J., Fabre H. (2012). Capillary electrophoresis for the assay of fixed-dose combination tablets of artesunate and amodiaquine. Malar. J..

[19] Lamalle C., Marini R.D., Debrus B., Lebrun P., Crommen J., Hubert P., Servais A., Fillet M. (2012). Development of a generic micellar electrokinetic chromatography method for the separation of 15 antimalarial drugs as a tool to detect medicine counterfeiting. Electrophoresis.

[20] Amin N.C., Fabre H., Blanchin M.D., Montels J., Aké M. (2013). Determination of artemether and lumefantrine in anti-malarial fixed-dose combination tablets by microemulsion electrokinetic chromatography with short-end injection procedure. Malar. J..

[21] Mufusama J., Hoellein L., Feineis D., Holzgrabe U., Bringmann G. (2018). Capillary zone electrophoresis for the determination of amodiaquine and three of its synthetic impurities in pharmaceutical formulations. Electrophoresis.

[22] De Souza J.C.Q., Chellini P.R., De Souza M.V.N., De Oliveira M.A.L. (2024). Primaquine analysis in pharmaceutical formulation using multiple and short-end injections by capillary zone electrophoresis–ultraviolet detection. J. Sep. Sci..

[23] Nagy C., Pesti A., Andrási M., Vasas G., Gáspár A. (2021). Determination of artemisinin and its analogs in *Artemisia annua* extracts by capillary electrophoresis–mass spectrometry. J. Pharm. Biomed. Anal..

[24] Rasmussen H.T., Goebel L.K., McNair H.M. (1991). Optimization of resolution in micellar electrokinetic chromatography. J. High Resolut. Chromatogr..

[25] Dehouck P., Van Schepdael A., Roets E., Hoogmartens J. (2001). Analysis of clindamycin by micellar electrokinetic chromatography with a mixed micellar system. J. Chromatogr. A.

[26] Krebs F., Zagst H., Stein M., Ratih R., Minkner R., Olabi M., Hartung S., Scheller C., Lapizco-Encinas B.H., Sänger-van de Griend C. (2023). Strategies for capillary electrophoresis: Method development and validation for pharmaceutical and biological applications—Updated and completely revised edition. Electrophoresis.

[27] Ruiz-Angel M.J., Peris-García E., García-Alvarez-Coque M.C. (2015). Reversed-phase liquid chromatography with mixed micellar mobile phases of Brij-35 and sodium dodecyl sulphate: A method for the analysis of basic compounds. Green Chem..

[28] Ahuja E.S., Foley J.P. (1995). Influence of dodecyl sulfate counterion on efficiency, selectivity, retention, elution range, and resolution in micellar electrokinetic chromatography. Anal. Chem..

[29] United States Pharmacopeial Convention (2018). United States Pharmacopeia–National Formulary (USP 41–NF 36).

[30] International Council for Harmonisation (2023). ICH Harmonised Guideline Q2(R2): Validation of Analytical Procedures.

